# Non-Invasive Preimplantation Genetic Testing: Cell-Free DNA Detection in Embryo Culture Media Using a Plasmonic Biosensor

**DOI:** 10.1021/acs.analchem.5c03164

**Published:** 2025-08-28

**Authors:** Noemi Bellassai, Anil Biricik, Matteo Surdo, Veronica Bianchi, Roberta D’Agata, Giulia Breveglieri, Roberto Gambari, Francesca Spinella, Giuseppe Spoto

**Affiliations:** † Department of Chemical Sciences, 9298 University of Catania, Viale Andrea Doria 6, 95125 Catania, Italy; ‡ INBB, Istituto Nazionale di Biostrutture e Biosistemi, Via dei Carpegna 19, 00165 Rome, Italy; § Eurofins Genoma Group, Via di Castel Giubileo, 62, 00138 Rome, Italy; ∥ Policlinico Città di Udine, Viale Venezia, 410, 33100 Udine, Italy; ⊥ Center “Chiara Gemmo and Elio Zago” for the Research on Thalassemia, Ferrara University, Via Luigi Borsari 46, 44121 Ferrara, Italy; # Department of Life Sciences and Biotechnology, Ferrara University, Via Luigi Borsari 46, 44121 Ferrara, Italy; ∇ UniCamillus, International Medical University, Via di Sant’Alessandro 8, 00131 Rome, Italy

## Abstract

In vitro fertilization (IVF) faces challenges in evaluating embryo quality and in determining the genetic health of embryos. Key biomarkers in the culture medium, including nucleic acids and proteins, offer promising avenues for noninvasive assessment. However, small sample volumes, low biomolecule concentrations, and potential contaminants complicate the reliable detection of genetic indicators. In this context, we developed a noninvasive preimplantation genetic testing (niPGT) approach using a superparamagnetic particles-enhanced surface plasmon resonance (SPR) imaging biosensor capable of detecting single-point mutations in nonamplified cell-free DNA released into the spent culture medium during early embryo development. Magnetic beads with a biotinylated locked nucleic acid sequence capture target sequences with single-nucleotide variations in the β-globin gene related to β-thalassemia. The assay discriminates between normal and mutated cell-free DNA evaluating their hybridization with peptide-nucleic acid probes. Our assay detects heterozygous or homozygous mutated DNA in spiked medium blank and spent culture medium at attomolar levels (1.5 pg μL^–1^, ∼0.75 aM) with minimal manipulation and no dilution. Only 10 μL of sample volume is needed for each analysis, providing reliable results within 2 h. This plasmonic-based test enables a rapid, biopsy-free evaluation of the embryo’s genetic status, helping to identify unaffected embryos with greater confidence and supporting informed selection for implantation.

## Graphical abstract

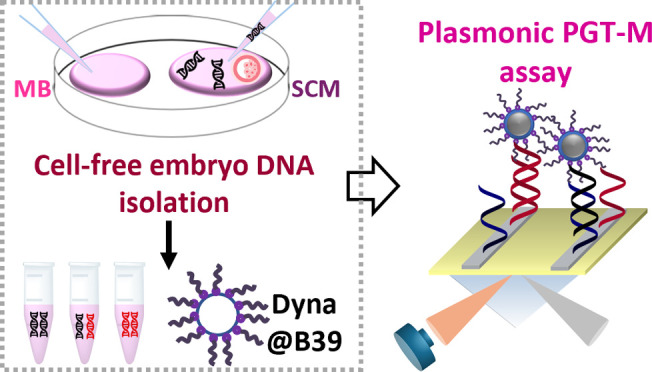

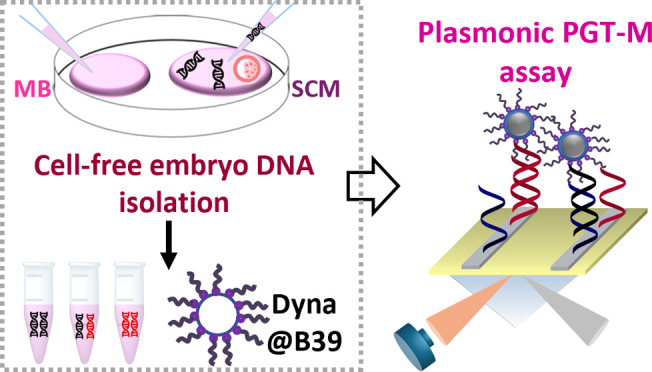

## Introduction

In vitro fertilization (IVF) is the most prevalent form of assisted reproductive technology (ART) to help millions of infertile couples worldwide in childbirth.^1–3^ A critical aspect of IVF treatment is the embryos’ properties, depending on various morphological and molecular parameters for transfer and effective implantation in ART cycles. Pregestational or preimplantation genetic testing (PGT) for monogenic disorders (PGT-M) is used to identify specific monogenic mutations in IVF embryos before their transfer to the mother, allowing individuals with hereditary diseases in their family to avoid transmitting them to their children. This procedure allows the selection of healthy embryos from genetically at-risk couples before implantation, thus increasing the chances of healthy pregnancies. PGT-M analyzes genetic defects on IVF embryos by using an invasive and labor-intensive approach due to blastomere or trophectoderm biopsies, which remove cells that form the embryo, potentially impacting the implantation of the embryos,^4^ pregnancy rates,^5^ and fetal development.^6^ New noninvasive PGT methods for analyzing cell-free DNA (cfDNA) in blastocoel fluid or spent culture medium (SCM) offer new approaches to assess embryo health, development, and implantation potential.^7–9^ Among the fluid-based biomarkers, cfDNA in SCM enables the analysis of the chromosomal/genetic status of the developing embryos,^10–15^ by improving PGT cost-efficiency and safety.^16–18^ However, the low amount of cfDNA, about 58–67 pg in 20 μL of embryo SCM in 3–6 days (2.90–3.35 pg μL^–1^ or 827–955 copies mL^–1^),^19^ demands more accurate and sensitive noninvasive approaches, including enhancing DNA collection and amplification methods and refining downstream analysis techniques. Nucleic acid amplification methods are commonly required to detect copy number variation and single nucleotide variations (SNVs).^7,20,21^ High error rates in target enrichment from DNA composition and nucleotide bias make these methods challenging to detect specific genetic variations. To reduce contamination from maternal DNA in residual cumulus cells, which can affect the embryo’s genetic composition, the procedure requires washing and replacing with fresh culture medium on day four of fertilization.^7^ Short cfDNA fragments from apoptotic cells also degrade quickly.^22^ Consequently, noninvasive PGT requires a certain amount of time in embryo culture (24–48 h) to ensure consistent cfDNA amounts in collected samples and requires immediate freezing of collected samples until processing.

The direct analysis of unamplified cfDNA in embryo media requires innovative strategies to improve SCM-based PGT diagnostic efficiency and clinical implementation. Surface plasmon resonance (SPR) technologies are promising optical biosensors that allow label-free, real-time target detection with high analytical performance.^23–25^ This plasmonic platform provides a versatile, efficient, and functional surface structure that enables reliable analyte measurements, particularly in biological fluids.^26^ Metallic nanoparticles enhance plasmonic biosensing, enabling ultrasensitive detection of cfDNA in human plasma samples.^27^ We reported on an SPR imaging (SPRI) assay that utilizes gold nanoparticles, and peptide nucleic acid (PNA) probes for ultrasensitive detection of unamplified genomic DNA from healthy individuals and homozygous or heterozygous β-thalassemia patients.^28^ Recently, we combined superparamagnetic beads with SPRI to directly detect SNVs in circulating tumor DNA from plasma samples of colorectal cancer patients.^29^ The inherent properties of the beads^30–32^ enhance bioassay performance by minimizing sample treatment, eliminating target amplification, speeding up turnaround time, and reducing sample volume needed for the analysis.

Here, we present a noninvasive method for preimplantation genetic screening of cell-free embryo DNA (cfeDNA) to detect single-nucleotide polymorphisms (SNPs) in spent embryo culture medium using a superparamagnetic particle-based SPRI sensing strategy. In particular, we detect homozygous and heterozygous mutations in the β^0^39-globin gene linked to β-thalassemia disease. Streptavidin-coated beads (1 μm in diameter) modified with a biotinylated oligonucleotide allowed the direct capture of cfeDNA from the spent culture medium, whereas two PNA probes could recognize wild-type, β^0^39 homozygous, and β^0^39 heterozygous nonamplified DNAs captured by the modified beads. The ability of cfeDNA discrimination arises from the combination of PNA/cfeDNA recognition and bead-enhanced SPRI detection. The new approach notably streamlines the preanalytical workflow compared to currently adopted protocols.^33^ To simulate the real sample, we first tested the detection of genomic DNA (gDNA) at 1.5 pg μL^–1^ (less than 450 copies mL^–1^) bearing normal and β^0^39 hetero/homo mutation spiked in the medium blank (MB). The plasmonic assay was then applied for cfeDNA detection in SCM from embryo cell cultures. The proposed plasmonic assay requires only 10 μL of sample volume and allows decreasing the readout and analysis times to under 2 h. Additionally, combining a microfluidic device with controlled stopping and restarting of the pumping system significantly reduced the injection volume for the plasmonic assay to 10 μL, eliminating the need for sample dilution.

## Experimental Section

### Functionalization of Superparamagnetic Particles

We dispersed Dynabeads MyOne Streptavidin C1 beads in washing buffer 1× obtained by diluting 2× binding and washing (B&W) buffer (10 mM Trizma hydrochloride solution pH 7.5, 1 mM EDTA, 2 M NaCl). Next, the beads were placed on a magnet for 2 min, and the supernatant was discarded. After three washing steps, the beads were resuspended in B&W buffer to twice their original volume. The beads were then incubated with biotinylated locked nucleid acids, LNAB39 (sequence 5′→3′: A+G+CA+G+C+C+T+A+AG/3BioTEG/, *T*_m_ = 63.3 °C, the bolded plus letters highlight the locked bases in LNA), by maintaining a specific LNAB39:beads ratio to ensure an optimal binding capacity (500 pmol of LNAB39 for 1 mg of beads). The incubation was performed for 15 min at room temperature using a HulaMixer Sample Mixer with gentle tilting and rotation (100 rpm, 40° as tilting angle). Dynabeads MyOne Streptavidin C1 beads decorated with LNAB39 (Dyna@B39) were placed in a magnet for 2 min to remove unbound oligonucleotides. The supernatant was collected to estimate the loading efficiency of the reaction. Lastly, Dyna@B39 pellet was washed three times with 1× washing buffer and resuspended in PBS buffer at 10 mg mL^–1^ as the stock solution. Both bare (Dynabeads MyOne Streptavidin C1) and conjugated Dyna@B39 beads were characterized at a concentration of 0.1 mg mL^–1^ in nuclease-free water by using Dynamic Light Scattering and ζ-potential measurements (Zetasizer Nano ZS ZEN3600, Malvern Instruments, Malvern, U.K.) (Table 1).

**Table 1:** Properties of Bare Dynabeads and Dyna@B39 Used for cfeDNA Detection by SPR^a^

	ξ ± sd (mV)	Z-aver ± sd (nm)	PDI ± sd	L.E. (%)
Dynabeads (*n* = 2)	–44.5 ± 0.7	1135 ± 32	0.18 ± 0.02	-
Dyna@B39 (*n* = 9)	–39.3 ± 0.9	1175 ± 113	0.10 ± 0.05	96 ± 3

a DLS measurements have been performed with 0.1 mg mL^–1^ beads in nuclease-free water. Replicates of independent measurements are indicated (*n*). ζ = Zeta potential; Z-aver = Z-average radius; PDI = Polydispersity index from DLS measurements; L.E. = loading efficiency.

### Embryo Medium Blank and Spent Culture Medium Samples

After controlled ovarian stimulation and induction of ovulation, oocyte retrieval was performed from β-thalassemia carrier women. Denudation of surrounding cumulus cells was conducted before intracytoplasmic sperm injection (ICSI) to avoid any contamination derived from maternal cumulus cells. Normally fertilized embryos after ICSI were cultured individually in a continuous single culture medium-CSCM (Irvine Scientific) in 20 μL culture-droplets under mineral oil, and the culture medium was changed on day 4 morning after a careful rinse of the embryo in a CSCM droplet. Embryo culture was prolonged until day 5–6 in 20 μL fresh CSCM under mineral oil. The culture of embryos was continuously accompanied by an empty CSCM droplet of 20 μL volume in the same Petri dish as the blank control. The embryos which reached the fully expanded blastocyst stage on days 5–6 were then moved to a biopsy dish for the routine PGT biopsy procedure, and the SBM were collected in PCR tubes, covered with 30 μL mineral oil and stored at −20 °C until their processing. After thawing, the total volume of each medium (up to 20 μL) was aspirated under the mineral oil layer with a sterile fine tip (5–20 μL) filtered instrument. Immediately afterwards, it was transferred to a sterile test tube, and the next step was started. Analysis of SCM samples collected from culture media of the embryos by trophectoderm biopsy and minisequencing method for the genotyping of β^0^39 mutation was reported in Table S3. Unfortunately, homozygous embryos were not collected during the clinical recruitment process, making them unavailable for the SCM analysis with the superparamagnetic bead-based SPRI assay.

### SPRI Detection of Genomic DNA in Medium Blank Samples

MB added with HSA (5% v,v) was heated for 30 min at 37 °C to simulate the embryo culture medium. gDNA solutions of wild-type (WT/WT), β^0^39 heterozygous (β^0^39/WT), and β^0^39 homozygous (β^0^39/β^0^39) samples (Table S3) were spiked in MB added with HSA at room temperature, were fragmented by sonication (3 min, ELMA Transsonic T480/H-2) and vortexing (1 min, IKA Vortex GENIUS 3) and denatured by heating at 95 °C for 5 min. After these treatments, 10 μL of 1.5 pg μL^–1^ solutions of wild-type or β^0^39 heterozygous or β^0^39 homozygous gDNAs in MB added with HSA were incubated with 2.0 mg mL^–1^ of Dyna@B39 for an hour at 25 °C under shaking at 750 rpm (ThermoMixer C, Eppendorf, Hamburg, Germany) (Figure 1, Step 1). A concentration of 1.5 pg μL^–1^ corresponds to approximately 450 copies mL^–1^ or 0.75 aM of gDNA. This calculation assumes that the molecular weight of gDNA is 660 g mol^–1^ per base pair and that each gDNA molecule consists of 3.2 × 10^9^ base pairs. The beads with the catched gDNA (Dyna@B39-DNA) were washed three times and resuspended in 10 μL of PBS buffer before SPRI analysis (Figure 1, Step 2). Our SPRI assay exploits the parallel hybridization of Dyna@B39-DNA with two PNA probes (PNA-N or PNA-M) covalently bound to the SPRI sensor (Figure 1, Step 3). A procedure was designed to introduce only the 10 μL of the Dyna@B39-DNA dispersion available for SPRI detection into the SPRI microfluidic system using a peristaltic pump operating at 10 μL min^–1^ (45 s). Dyna@B39-DNA dispersion was pushed into the microfluidic device by pumping PBS buffer for 1 min and 15 s until the beads reached the PNA-modified gold sensor. We stopped the peristaltic pump for 20 min to acquire the SPRI signal, enabling parallel evaluation of hybridization between Dyna@B39-DNA and PNA probes. The unbound Dyna@B39-DNA were removed by washing the surface with PBS buffer (10 μL min^–1^ for 10 min). Data used for sample evaluations were obtained by considering a stable SPRI signal detected during the washing step at 2000 s under the same pumping conditions. We loaded wild-type and β^0^39 mutated gDNA samples into the microfluidic device, alternating sample order to prevent artifacts. For SCM analysis, we adopted the same protocols to fragment cfeDNA in SCM of patients with β^0^39 heterozygous mutation (cfeGra1M and cfeGra5M) and healthy donors (cfeGra2M, cfeGra3M, cfeGra4M, cfeSam1M, cfeSam2M, cfeSam3M, cfeSam4M, and cfeSam5M) (Table S3). Similarly, we captured cfeDNA in SCM samples using Dyna@B39 (Figure 1, Step 1), and detected the target mutation by hybridizing Dyna@B39-DNA bearing cfeDNA with the PNA probes (Figure 1, Step 3). SPRI curves recorded when detecting β^0^39 heterozygous cfeDNA, wild-type cfeDNA and spiked samples with β^0^39 homozygous gDNA from patients and healthy donors are shown in Figures S3 and S4.

**Figure 1:**
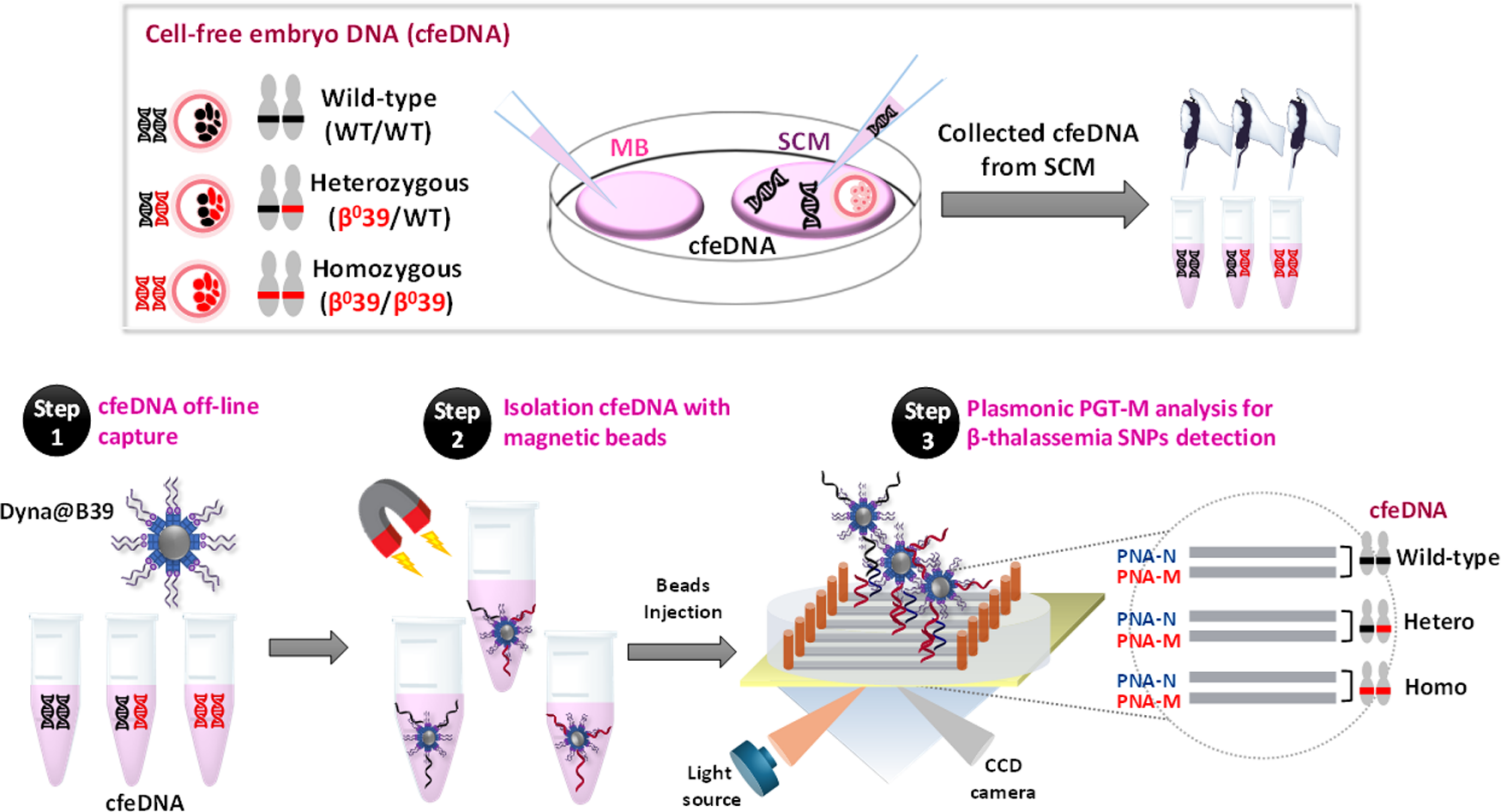
A pictorial description of the PGT-M superparamagnetic particle-based plasmonic assay for detecting β^0^39 SNPs linked to β-thalassemia in cfeDNA released in SCM is shown. We captured cfeDNA in SCM sample using superparamagnetic beads modified with a biotinylated locked nucleic acid probe (Dyna@B39) (Step 1). Control experiments used MB without cfeDNA. Subsequently, beads bearing the catched DNA (Dyna@B39-DNA) were isolated (Step 2). After the parallel immobilization of normal and mutated PNA probes (PNA-N and PNA-M) on the SPRI sensor surface, we discriminated between wild-type and β^0^39 homozygous, or β^0^39 heterozygous cfeDNAs during the interaction of Dyna@B39-DNA with PNA probes (Step 3).

## Results and Discussion

### Plasmonic PGT-M Assay Design for SNPs Detection in cfeDNA by using Magnetic Particles

Figure 1 shows a pictorial description of PGT-M superparamagnetic particle-based plasmonic assay for the detection of β^0^39 SNPs cfeDNA and wild-type cfeDNA released in SCM.

We decorated superparamagnetic beads with a biotinylated locked nucleic acid probe sequence (LNAB39) that captures wild-type, homozygous or heterozygous β^0^39 mutated DNAs because it is complementary to a shared sequence in the human β-globin gene. Hydrodynamic diameter and ζ-potential of the beads with and without LNAB39 (Dyna@B39) are presented in Table 1. Dyna@B39 captured cfeDNA in SCM samples (10–20 μL) by hybridizing the shared sequence (Figure 1, Step 1). The resulting beads (Dyna@B39-DNA) were isolated by applying an external magnetic field, resuspended in 10 μL of PBS buffer (Figure 1, Step 2) and injected into two microchannels of the SPR apparatus corresponding to PNA-N and PNA-M probes modified surfaces (Figure 1, Step 3). We identified wild-type, β^0^39 homozygous, or β^0^39 heterozygous cfeDNA by comparing SPR signals recorded when the same Dyna@B39-DNA sample interacted with PNA-N and PNA-M probes (Figure 1, Step 3).

As previously reported,^28,29^ we immobilized PNA probes on a dithiobissuccinimidyl propionate (DTSP) modified gold surface through amine coupling with the N-terminus of probes. PNAs more effectively recognize single-base mismatches in DNA hybridization compared to other similar nucleic acid structures.^34–36^ For this reason, we designed two PNA probes which differ from a single nucleotide to discriminate SNPs in cfeDNA (Table S1). PNA-N recognizes the wild-type cfeDNA sequence, whereas PNA-M identifies β^0^39 mutation. We introduced PNA-N and PNA-M solutions (0.05 μM in PBS) for 20 min in adjacent microchannels in contact with the DTSP-modified gold surface. This configuration enables control over the surface density of PNAs, which typically influences the efficiency of the hybridization.^37^ The kinetic profiles for PNA-N and PNA-M parallel immobilizations show similar SPR profiles to those previously reported for similar systems (Figure S1a,b).^29^

### Plasmonic PGT-M Assay for SNPs Detection in Genomic DNA Spiked in Medium Blank

We tested our assay using wild-type (WT/WT), β^0^39 heterozygous (β^0^39/WT), and β^0^39 homozygous (β^0^39/β^0^39) genomic DNAs (gDNAs) extracted from the blood of healthy donors and β-thalassemia patients (Table S2).^28^ gDNAs were spiked in MB supplemented with 5% HSA. Spiked gDNA samples were used to simulate culture medium samples containing cfeDNA released during embryo development. We set the gDNA concentration at 1.5 pg μL^–1^ as the minimum DNA level in SCM during the embryo’s early development.^19,38–40^

Before capturing gDNA with 2.0 mg mL^–1^ Dyna@B39, we treated gDNA solutions to produce suitable shorter, single-stranded DNA sequences. The high melting temperature of LNA probe on Dyna@B39 supported hybridization with the unamplified gDNA by enhancing duplex stability and enabling efficient capture at lower temperatures.

Figure 2 shows the SPRI change in percent reflectivity (Δ%*R*) over time during the interaction of PNA probes with Dyna@B39-DNA bearing wild-type (Figure 2a), β^0^39 heterozygous (Figure 2b), or β^0^39 homozygous (Figure 2c) gDNAs. 10 μL of the Dyna@B39-DNA dispersion was injected into two adjacent microchannels and then propelled by PBS until it reached the PNA-N and PNA-M-modified gold sensor.

**Figure 2:**
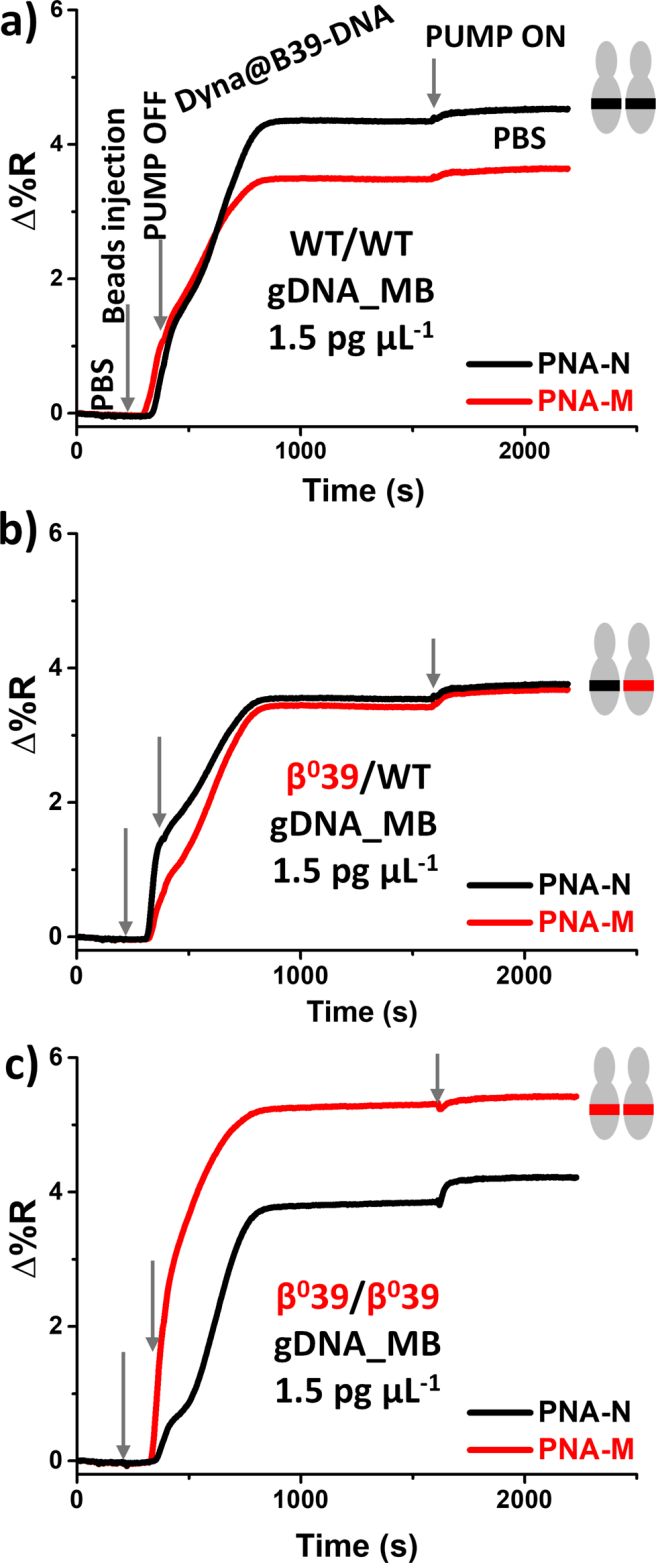
Representative time-dependent SPRI curves detected during the interaction between the surface-immobilized PNA-N and PNA-M probes and Dyna@B39-DNA beads resulting from the capture of 1.5 pg μL^–1^ (a) wild-type (WT/WT), (b) β^0^39 heterozygous gDNA (β^0^39/WT), and (c) β^0^39 homozygous (β^0^39/ β^0^39) gDNA spiked in MB supplemented with 5% HSA. The on and off operation of the pumping system caused irregular curve shapes.

After bead injection, the SPRI signal rapidly increases, indicating that Dyna@B39-DNA dispersion has reached the plasmonic sensor. The signal change allows us to monitor the position of the beads within the microchannels and then stop the continuous-flow fluidic system. Turning off the pumping system favors bead sedimentation and promotes the binding of Dyna@B39-DNA to the PNA probe. This results in a larger change in the plasmonic signal due to the high refractive index of the magnetic particles. Using this approach, a few microliters of Dyna@B39-DNA were introduced into microchannels and brought into contact with the PNA-modified surface for SNP detection via PNA/DNA hybridization. After reactivating the pump, unbound beads were displaced from the surface by creating slight variations in the SPRI signal. The minimum sample volume of 10 μL used per analysis prevents excessive dilution while effectively utilizing the typical 8 to 15 μL sample volume available from each embryo culture, thereby improving the assay sensitivity.

SPRI responses we observed aligned perfectly with our expectations. Dyna@B39-DNA derived from 1.5 pg μL^–1^ WT gDNA samples preferentially interacted with PNA-N over PNA-M (Figure 2a). Dyna@B39-DNA derived from β^0^39 heterozygous gDNA, brings an equal amount of WT and β^0^39 mutated sequences, yielding similar SPRI responses from both PNA-N and PNA-M (Figure 2b). In contrast, Dyna@B39-DNA derived from 1.5 pg μL^–1^ β^0^39 homozygous gDNA samples showed a stronger interaction with PNA-M (Figure 2c). The SPRI response evaluation of the Dyna@B39-DNA interaction with the noncomplementary PNA probe helped us assess the differential response due to a single mismatch between the PNA-N and PNA-M probes. In each experiment, we employed a negative control by comparing plasmonic signals from the same DNA sample interacting with two different PNA probes, differing by just one nucleotide. This approach allowed us to verify the assay’s selectivity in discriminating target sequences, as demonstrated by the signals from the parallel interactions with complementary and noncomplementary PNA probes being opposite.

We calculated the ratio of Δ%*R* values at 2000 s during rinsing after hybridizing the Dyna@B39-DNA dispersion with both PNA probes. Figure 3 shows the ratio of Δ%*R* values detected when the same Dyna@B39-DNA sample interacted with PNA-M (Δ%*R*_PNA‑M_) and PNA-N (Δ%*R*_PNA‑N_) probes. Data shown were obtained from replicated independent experiments aimed at detecting 1.5 pg μL^–1^ WT, β^0^39 hetero, or β^0^39 homo gDNAs spiked in MB supplemented with 5% HSA.

**Figure 3:**
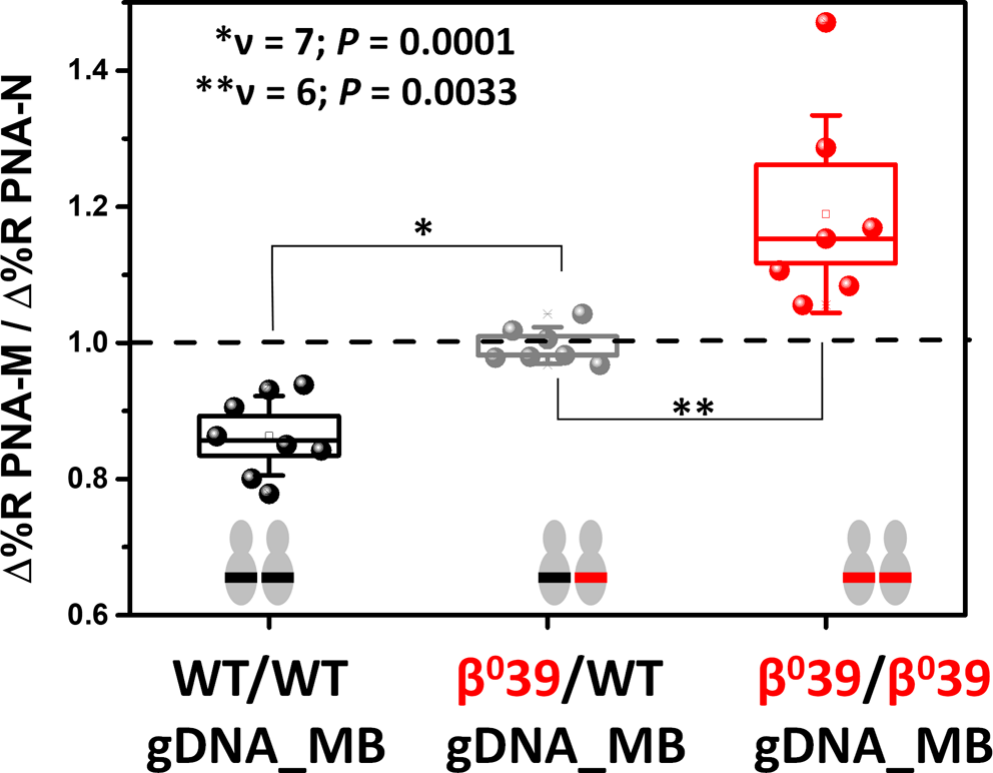
Δ%*R*_PNA‑M_/Δ%*R*_PNA‑N_ values from replicated experiments aimed at detecting wild-type, β^0^39 heterozygous and β^0^39 homozygous gDNAs samples 1.5 pg μL^–1^ in MB added with 5% HSA. Δ%*R* values were taken after 2000 s of hybridization between Dyna@B39-DNA and PNA probes, with the dashed line indicating a Δ%*R*_PNA‑M_/Δ%*R*_PNA‑N_ value of 1.

The population mean value of Δ%*R*_PNA‑M_/Δ%*R*_PNA‑N_ ratios calculated after SPRI analysis of 1.5 pg μL^–1^ mutated β^0^39 homo gDNA samples (population mean confidence interval at the 95% level for the ratio CI = 1.19 ± 0.14, *n* = 7) was different from that of β^0^39 hetero gDNA samples (Δ%*R*_PNA‑M_/Δ%*R*_PNA‑N_ 95% CI = 0.99 ± 0.02, *n* = 7. Two-tailed *t* test, level 95%, *p*-value = 3.3 × 10^–3^). In addition, the difference in population mean value of Δ%*R*_PNA‑M_/Δ%*R*_PNA‑N_ ratios obtained by SPRI analysis of 1.5 pg μL^–1^ mutated β^0^39 hetero gDNA samples was significantly different from that of wild-type gDNA samples (Δ%*R*_PNA‑M_/Δ%*R*_PNA‑N_ 95% CI = 0.86 ± 0.06, *n* = 8. Two-tailed *t* test, level 95%, *p*-value = 1.0 × 10^–4^). Interestingly, the distribution of data from replicate analyses of wild-type (*s* ^2^ = 0.0034), β^0^39 hetero (*s* ^2^ = 0.0007) and β^0^39 homo (*s* ^2^ = 0.0210) gDNA samples is characterized by different variances. Specifically, the population variances for the wild-type and β^0^39 hetero gDNAs can be considered equal (*F*-test, α = 0.05, *p*-value = 0.07743). In contrast, the population variances for the wild-type and β^0^39 homo gDNAs are different (*F*-test, α = 0.05, *p*-value = 0.03023), as are the population variances for the β^0^39 hetero and β^0^39 homo gDNAs (*F*-test, α = 0.05, *p*-value = 0.00069). The β-globin gene is well-known for its high level of polymorphism, meaning it has many variations in its DNA sequence. Specifically, the presence of different secondary structures in DNA fragments, potentially caused by polymorphisms near the β^0^39 mutation, could negatively impact the folding and interaction of DNA fragments, explaining some of the variability observed in the experimental results.^41^ Another peculiar aspect is the reduced variability of data for β^0^39 hetero gDNA samples. Such an aspect aligns with previous findings when analyzing wild-type, β^0^39 hetero, and β^0^39 homo gDNA samples in PBS buffer using a different SPRI assay, and is justified by considering that only β^0^39 hetero gDNA provides a specific interaction with both PNA-N and PNA-M probes.^28^ When detecting wild-type or β^0^39 homo gDNA samples, only one of the PNA probes is expected to specifically bind to the complementary sequence in the target. The SPRI signal measured from the other PNA probe results solely from nonspecific interactions, which can contribute to increased signal variability.

As already mentioned,^29,42,43^ we achieved attomolar concentrated DNA detection by integrating off-line target capture using functionalized superparamagnetic beads, followed by beads adsorption on the sensor surface and specific interaction with PNA probes. The experiments presented here demonstrate that the SPRI assay effectively detects spiked samples with gDNA at a concentration of 1.5 pg μL^–1^. The Dyna@B39 used to capture the target gDNA from the spiked medium blank exposes a total of a few hundred picomoles of LNAB39 probes. This amount is many orders of magnitude greater than the number of target molecules to be captured from each sample. Such conditions are expected to enhance target capture by Dyna@B39, as they shift the chemical equilibrium in favor of complex formation. The efficiency of LNAB39 probes on the beads’ surface, combined with the beads’ slow sedimentation rate, ensures the effective capture of DNA targets directly from complex media, eliminating the need for sample dilution or manipulation. The polymeric shell of the beads reduces nonspecific adsorption of interfering biomolecules by preventing beads aggregation in human plasma.^44^ As shown for single magnetic domain-induced superparamagnetic particle aggregation,^45–47^ the clustering of magnetic beads during specific hybridization between the modified beads and the PNA probe locally modifies dielectric constant of the sensor surface, enhancing plasmonic detection.

To assess the applicability of detecting low levels of cfeDNA for a noninvasive preimplantation genetic testing (niPGT) approach, we established a calibration curve across a dynamic range of target concentrations (0.5–20.0 pg μL^–1^) of wild-type (WT/WT) gDNA spiked into the medium blank, utilizing the magnetic bead-based plasmonic assay (see Figure S2). We determined the minimum detection concentration (MDC) and the reliable detection limit (RDL) as key analytical parameters of the assay, which were found to be 0.41 and 0.73 pg μL^–1^, respectively. This was achieved using a four-parameter logistic curve fitting procedure.^48,49^

### Plasmonic PGT-M Assay for SNPs Detection in cfeDNA Circulating in Spent Culture Medium of Embryos

We conducted SPR experiments to detect SNPs in cfeDNA released by embryos in SCM to validate the assay against real samples. SCM samples collected from culture media of the embryos were previously diagnosed by trophectoderm biopsy and minisequencing method for the genotyping of β^0^39 mutation (Table S3). The detection of SNV in mutated β^0^39 heterozygous and wild-type cfeDNA involved the sampling of SCM. The available SCM sample volume ranged from 8 to 15 μL, collected during embryo development. We fragmented cfeDNA from patients’ embryo cultures and directly captured it by adding 2.0 mg mL^–1^ of Dyna@B39 to the SCM sample. We adjusted the incubation volume, which contained only cfeDNA in SCM with magnetic beads, to a final volume of 10 or 20 μL, resulting in a minimal sample dilution (approximately 1.3 times). This approach allows for the direct analysis of SCM samples by injecting 10 μL into two adjacent microfluidic channels and measuring two replicates of the same sample simultaneously when the incubation volume is 20 μL. To evaluate the effectiveness of the plasmonic assay in identifying SNPs, we performed parallel detection of β^0^39 mutated homozygous gDNA spiked in MB at final 1.5 pg μL^–1^ concentration in 10 μL, while simultaneously analyzing cfeDNA in SCM.

Figure 4 displays the Δ%*R*_PNA‑M_/Δ%*R*_PNA‑N_ ratios obtained from replicated independent experiments involving cfeDNA in SCM from embryo cultures of seven donors without β^0^39 mutations (samples ID: cfeSam1M, cfeSam2M, cfeSam3M, cfeSam4M, cfeSam5M, cfeGra2M, and cfeGra3M). Additionally, it includes two patients’ embryo cultures with heterozygous β^0^39 mutated cfeDNA (samples ID: cfeGra1M and cfeGra5M), and three control MB samples spiked with homozygous β^0^39 mutated gDNA (gDNA samples ID: Fe6_MB, pt#4_MB, and Fe77_MB) (Table S4). SPRI kinetic curves from the analyses mentioned above are presented in the Supporting Information (Figures S3 and S4).

**Figure 4:**
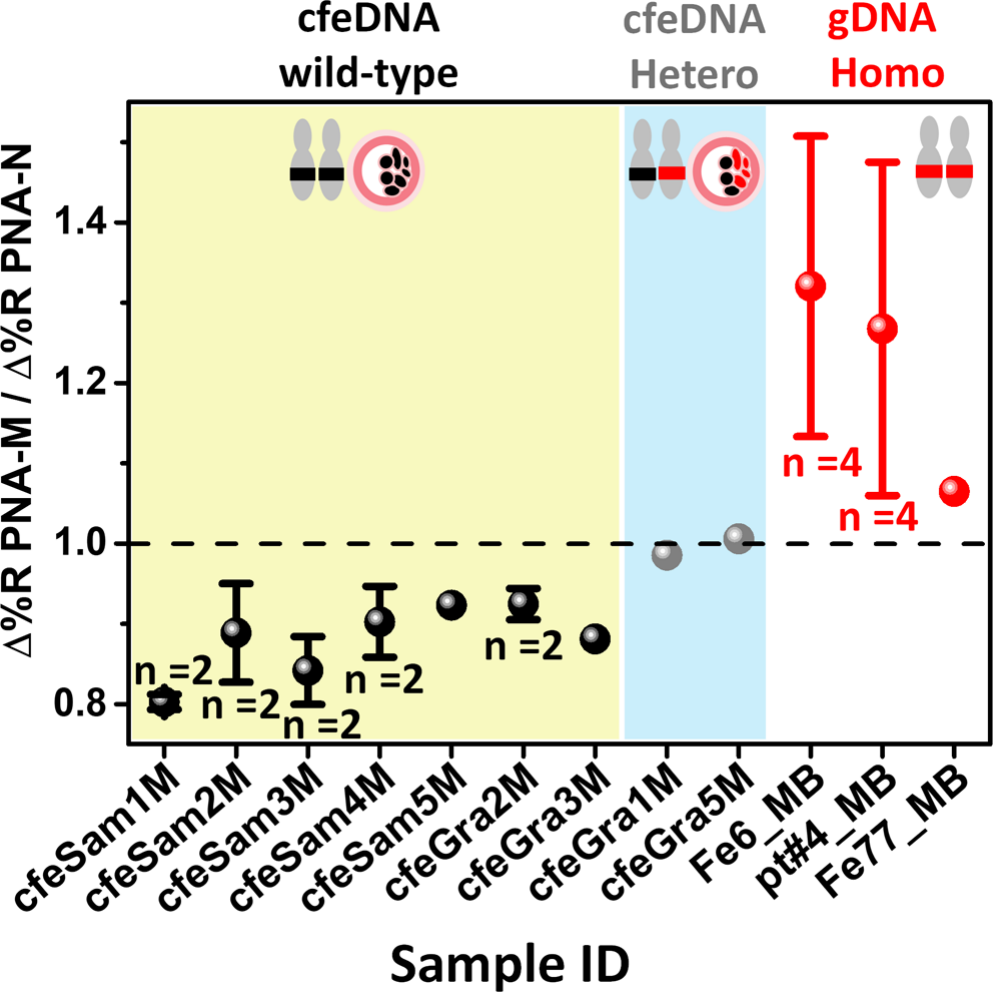
Δ%*R*_PNA‑M_/Δ%*R*_PNA‑N_ ratios referring to SPR detection of Dyna@B39-DNA obtained from patients’ embryo, healthy donors and gDNA spiked. cfeDNA in SCM from seven donors’ embryo cultures without β^0^39 mutations (wild-type, WT/WT) (sample ID: cfeSam1M, cfeSam2M, cfeSam3M, cfeSam4M, cfeSam5M, cfeGra2M, and cfeGra3M) showed ratio values less than 1. In contrast, cfeDNA in SCM with heterozygous β^0^39 (β^0^39/WT) from two patients’ embryo cultures (samples ID: cfeGra1M and cfeGra5M) yielded ratio values close to 1. Nine MB added with 5% HSA samples spiked with β^0^39 homozygous mutated gDNA (β^0^39/ β^0^39) (sample ID: Fe6_MB, pt#4_MB, Fe77_MB) showed ratio values greater than 1. The number of replicates (*n*) is indicated. Samples cfeSam5M, cfeGra3M, cfeGra1M, and cfeGra5M were not replicated due to limitations in sample size. Error bars represent the standard deviation of the replicated measurements.

The analyses showed that normal (WT/WT) cfeDNA samples in SCM had Δ%*R* ratios below 1, while cfeDNA with the β^0^39 heterozygous mutation (β^0^39/WT) had Δ%*R*_PNA‑M_/Δ%*R*_PNA‑N_ ratios around 1, confirming the assay’s ability to differentiate normal embryos from β-thalassemia carriers. Additionally, MB with 5% HSA samples spiked with β^0^39 homozygous mutated (β^0^39/β^0^39) gDNA produced ratios above 1. These results confirm the expected preferential adsorption of Dyna@B39-DNA carrying β^0^39/β^0^39 homozygous DNA on the complementary PNA-M probe, while a favored interaction with PNA-N probe is observed for WT/WT normal cfeDNA samples. This evidence provides a clearer framework for comparing traditional PCR methods with the SPRI PCR-free assay used for noninvasive preimplantation genetic testing in SCM for monogenic diseases. The proposed assay detects cfeDNA in the spent culture medium of embryos after a straightforward preanalytical processing. The three-step workflow consists of the preanalytical treatment of cfeDNA, the capture of cfeDNA by Dyna@B39, and the interaction of Dyna@B39-DNA with PNA-M and PNA-N probes. The overall turnaround time is only 100 min, which includes DNA fragmentation and denaturation (10 min), cfeDNA capture by Dyna@B39 (60 min), and SPRI detection of the parallel hybridization of Dyna@B39-DNA with PNA-M and PNA-N probes (30 min). In contrast to quantitative PCR-based methods, which can require more than 100 min for analysis,^27,50^ the SPRI assay streamlines the analytical workflow and shortens the duration of the experiment. Compared to other magnetic beads-based plasmonic assays,^29^ the new assay halves the analysis volume to 10 μL with no DNA target dilution and amplification. We modified magnetic beads with an LNA probe to enhance the stability of duplexes formed with target DNA. Compared to metal nanoparticles utilized to enhance SPR signals,^28^ superparamagnetic beads directly capture cfeDNA from biofluids and allow easy separation of the beads and target enrichment via an external magnetic field. The new SPRI assay correctly identified wild-type over heterozygous SNPs related to β-thalassemia in cfeDNA in SCM samples after collection from day 4 to day 6 from IVF embryos. Therefore, it could represent a convenient alternative approach for noninvasive preimplantation genetic testing for monogenic diseases for the characterization of DNA polymorphisms associated with clinical response to drug treatment in pharmacogenomics-based precision medicine protocols.^51^

## Conclusions

In contemporary clinical practice, there is a notable absence of noninvasive effective methodologies for evaluating monogenic diseases and chromosomal asset of the embryos during IVF prior to implantation. The embryo secretome, which includes nucleic acids and proteins released into the culture medium, is crucial for assessing embryo quality through a noninvasive PGT (niPGT) approach. The discovery of cfeDNA in SCM represents a significant advancement in ART, allowing for evaluating embryo quality based on genetic status without resorting to invasive techniques.

The proposed study is the application of the SPRI assay using superparamagnetic beads to capture complementary DNA sequences directly in SCM, thereby improving the detection of β^0^39 SNVs mutations both in homo and heterozygous configurations. This is achieved by hybridizing Dyna@B39, which carries target DNA with complementary and mismatched PNA probes simultaneously. Following the off-line capture of DNA sequences by Dyna@B39 (60 min), the hybridization reaction between Dyna@B39-DNA, which contains the captured target, and complementary and noncomplementary PNA probes immobilized on the plasmonic biosensor facilitates the detection of SNVs by recording real-time SPRI responses. The off-line DNA capture by the beads reduces nonspecific adsorption of unwanted biomolecules from SCM, which typically interfere with analyte detection, thus achieving ultrasensitive SNV detection. The combined effect of beads in enhancing plasmonic signals and the unique properties of PNA probes enabled the detection of fewer than 450 copies mL^–1^ of β^0^39 hetero/homo DNA and differentiation between mutated and wild-type samples using a sample volume of only 10 μL per analysis. This method eliminates several complex steps in analyzing SCM samples from embryo cultures, such sample extraction and purification followed by targeted or whole genomic amplification. The time required for preanalytical sample treatment is significantly reduced to 10 min, with the total turnaround time being less than 2 h (only 100 min). To conclude, the SPRI biosensing approach enhanced by superparamagnetic particles allows for the swift, direct detection of monogenic-derived sequences in SCM without PCR amplification. This represents a significant advancement in noninvasive PGT methods for assessing embryo genetic condition before potential implantation and offers new opportunities for applications in early cancer detection or pharmacogenomics-based medical intervention.

## Supplementary Material



## Data Availability

All data needed to evaluate the conclusions in the paper are present in the paper and/or the Supporting Information.

## Notes

Description of PNA probe sequences; Quantification of genomic DNA sequences by PCR amplification from blood β-thalassemic patients; Analysis of SCM samples collected from culture media of the embryos by trophectoderm biopsy and minisequencing method; SPR kinetic curves of PNA probe immobilization; Calibration curve; SPR experimental curves recorded during cfeDNA detection in SCM samples (PDF)
