# Supplementary material for: Non-Invasive Preimplantation Genetic Testing: Cell-Free DNA Detection in Embryo Culture Media Using a Plasmonic Biosensor
Source: Anal Chem. 2025 Aug 28;97(35):19241–8. doi: 10.1021/acs.analchem.5c03164 (PMC12424020; doi:10.1021/acs.analchem.5c03164)
Supplement: Supplementary file 1 [file ac5c03164_si_001.pdf]

Supporting Information

Non-Invasive Preimplantation Genetic Testing: Cell-Free DNA Detection in Embryo Culture Media Using a Plasmonic Biosensor

Noemi Bellassai[1,2], Anil Biricik[3], Matteo Surdo[3], Veronica Bianchi[4], Roberta D'Agata[1,2], Giulia Breveglieri[5], Roberto Gambari[5,6], Francesca Spinella[3,7], Giuseppe Spoto[1,2]*

[1]Department of Chemical Sciences, University of Catania, Viale Andrea Doria 6, 95125, Catania, Italy.

[2] INBB, Istituto Nazionale di Biostrutture e Biosistemi, Via dei Carpegna 19, 00165, Rome, Italy.

[3] Eurofins Genoma Group, Via di Castel Giubileo, 62, 00138, Rome, Italy.

[4] Policlinico Città di Udine, Viale Venezia, 410, 33100, Udine, Italy.

[5] Department of Life Sciences and Biotechnology, Ferrara University, Via Luigi Borsari 46, 44121, Ferrara, Italy.

[6] Center "Chiara Gemmo and Elio Zago" for the Research on Thalassemia, Ferrara University, Via Luigi Borsari 46, 44121, Ferrara, Italy.

[7] UniCamillus, International Medical University, Via di Sant'Alessandro 8, 00131, Rome, Italy.

*Corresponding author: giuseppe.spoto@unict.it

Table of contents

Table S1. Sequences and acronyms of PNA probes used for SPR experiments.

Table S2. Quantification of genomic DNA sequences by PCR amplification from blood beta-thalassemic patients using for Dyna@B39-based plasmonic assay.

Table S3. Analysis of SCM samples collected from culture media of the embryos by trophectoderm biopsy and minisequencing method for the genotyping of $\beta^0$39 mutation.

Table S4. Population mean confidence intervals of $\Delta\%R_{PNA-M}/\Delta\%R_{PNA-N}$ ratios for analyzed SCM, with ID of samples used for the analysis and the number of replicates.

Figure S1. a) Δ%R over time detected for PNA-N or PNA-M probes multichannel parallel immobilization on DTSP-SAM functionalized gold surface.

Figure S2. (a) SPRI calibration curve of different WT/WT gDNA concentrations spiked in medium blank added with 5% v,v of HSA. (b) Four-parameter logistic function for experimental data fitting of SPRI calibration curve.

Figure S3. a) SPRI signals (Δ%R) over time for the hybridization of PNA-M and PNA-N probes with Dyna@B39-DNA bearing cfeDNA without $B^0$39 homo/hetero mutation (wild-type donors) captured in SCM of 5 embryos' cultures coming from the same parents. b) To compare the plasmonic signals, we performed the analysis of spiked $\beta^0$39 homo gDNA in MB plus 5% HSA in the same experiment.

Figure S4. a) SPRI signals (Δ%R) over time for the hybridization of PNA-M and PNA-N probes with Dyna@B39-DNA bearing cfeDNA without $\beta^0$39 homo/hetero mutation (wild-type donors) captured in SCM of 2 embryos' cultures coming from the same parents. b) To compare the plasmonic signals, we performed the analysis of spiked $\beta^0$39 homo gDNA in MB plus 5% HSA in the same experiment. c) SPRI signals (Δ%R) over time for the hybridization of PNA-M and PNA-N probes with Dyna@B39-DNA bearing cfeDNA with $\beta^0$39 hetero mutation captured in SCM of 2 embryos' cultures coming from the same parents.

Materials

Reagents were obtained from commercial suppliers and used without further purification. Phosphate buffered saline (PBS) solutions at pH 7.4 (137 mM NaCl, 2.7 mM KCl, phosphate buffer 10 mM) were obtained from VWR (Italy). Ethanol, dimethyl sulfoxide, dithio-bis-succinimidyl propionate (DTSP), sodium chloride (NaCl), ethylendiaminetetraacetic acid disodium salt dihydrate (Na_2EDTA), Trizma® hydrochloride solution, human serum albumin (HSA) were purchased from Sigma-Aldrich (Italy). PNA probe sequences for wild-type and β°39 mutated β-globin sequences were obtained from HLB PANAGENE Co., LTD. Dynabeads™ MyOne™ Streptavidin C1 beads were purchased by Thermo Fischer Scientific (Italy). Thermo Fisher and IDT Technologies provided the biotinylated oligonucleotides to functionalize magnetic particles. Irvine scientific continuous Single Culture-NX (CSCM-NX, Catalog ID 90167) as medium blank for embryo culture was purchased from FUJIFILM Italia S.p.A. Gold chips were purchased from Xantec bioanalytics GmbH (Germany). Ultra-pure water (Milli-Q Element, Millipore) was used for all the experiments.

Surface Plasmon Resonance Imaging (SPRI)

As detailed elsewhere,[1] we conducted SPRI experiments using an SPR imager apparatus (GWC Technologies, USA). We created a poly(dimethylsiloxane) (PDMS) microfluidic device featuring six parallel microchannels (80 μm depth, 1.31 cm length, 762 μm width). This device was adhered to the gold surface of the SPRI sensor chip using double-sided adhesive tape (Neschen Gudy ultra clear double-sided adhesive tape). The arrangement of the parallel microchannels enables up to six independent SPRI experiments to be performed simultaneously. We embedded PEEK tubes within the device to connect the microfluidic device to a peristaltic pump (IPC, Ismatec SA, Switzerland) and used Tygon tubes (UpChurch Scientific) for the connections. A refractive index matching liquid was employed to ensure optical contact between the gold chip and the prism. Data analysis was conducted using OriginPro 9.0 software from OriginLab Corporation, Northampton, USA. As mentioned previously,[1] a cleaning procedure was established for the fluidic system to reduce the risk of sample contamination and memory effects.

PNA probes

We designed PNA probes for wild-type (PNA-N) and β°39 mutated sequences (PNA-M) to get melting temperatures higher than 65°C (at 4 μM) through to an empirical model[2] using fully matched complementary DNA sequences. Two 2-(2-aminoethoxy)ethoxyacetic acid (AEEA) spacers were added at the N terminus of PNA probes to enhance the accessibility of the complementary DNA target

sequence, as already demonstrated using microarray technology.[3] As already reported by D'Agata et al., the acronyms and sequences of PNA probes are in Table S2.[4]

Table S1. Sequences and acronyms of PNA probes used for SPR experiments.

PNA probe sequences for β-thalassemia mutation			
Mutation	Sequence[a] (N-term → C-term)	PNA acronym	T_m (°C) calc.[b]
$\beta^0$39	$(AEEA)_2$ - CTCT**A**GGTCCAA	PNA-M	61.5
Wild-type	$(AEEA)_2$ - CTCTGGGTCCAA	PNA-N	66.0

[a]Bolded letter highlights the mutated base.

[b]PNA:DNA (with full match complementary DNA) melting temperatures, calculated for 4 µM concentration, according to previous work,[2] using the online available PNA design tool from PNA-BIO http://pnabio.com/support/PNA_Tool.htm).

Functionalization of SPRI gold chips with PNA probes

A bare gold chip was exposed to UV-ozone for 5 min, washed with ethanol for 10 min, and dried using a nitrogen stream. After cleaning, the sensor was soaked in DTSP solution (4 mM in DMSO) for 48 hours with constant and gentle agitation at 25°C. Prior to the SPRI experiments, the functionalized surface was rinsed with ethanol and attached to the microfluidic device using double-sided adhesive tape. PNA-N and PNA-M probes were anchored on the DTSP-modified gold sensor through an amine-coupling reaction between N-hydroxysuccinimidyl (NHS) ester ends of DTSP layer and the N-terminal group of $(AEEA)_2$ linker. We achieved spatially separated immobilization of the probes by injecting PNA solutions (0.05 µM in PBS, flow rate 10 µL min^{-1}) into the parallel channels of the microfluidic device over 20 minutes. The gold sensor was washed with PBS for 10 minutes (flow rate 10 µL min^{-1}) to eliminate unbound PNA molecules. To fully deactivate any unreacted NHS end groups of the DTSP layer, a solution of 0.5 M of Trizma® hydrochloride pH 8.0 was introduced in the microfluidic device for 10 minutes (10 µL min^{-1}). Finally, the PNA-modified sensor was washed with PBS buffer for 10 minutes.

The quantification of the thickness of the surface layer and the probe surface coverage, as described by Shumaker-Parry et al.,[5] has been calculated by exploiting SPRI signals as changes in per cent reflectivity (Δ%R) over time. We obtained 12 10^{12} molecules cm^{-2} as the surface density for both PNAs, using the refractive index and density of PNA molecules equal 1.4 and 1.2 g cm^{-3}, respectively (Fig. S1 a,b). We estimated a signal variability ranging from 18% to 20% (CV%) for PNA probes over 44 parallel performed immobilizations.

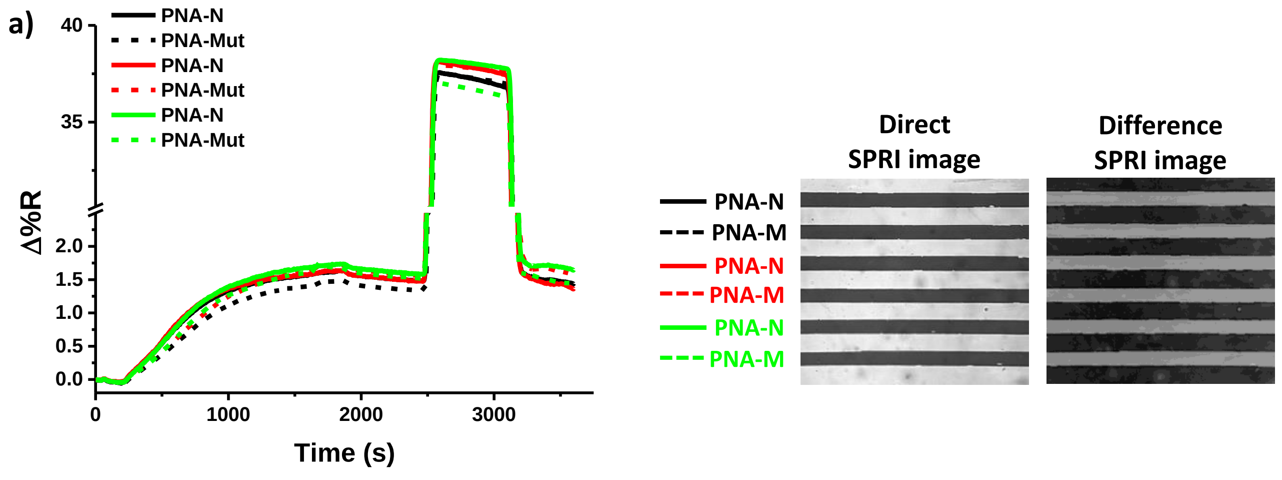

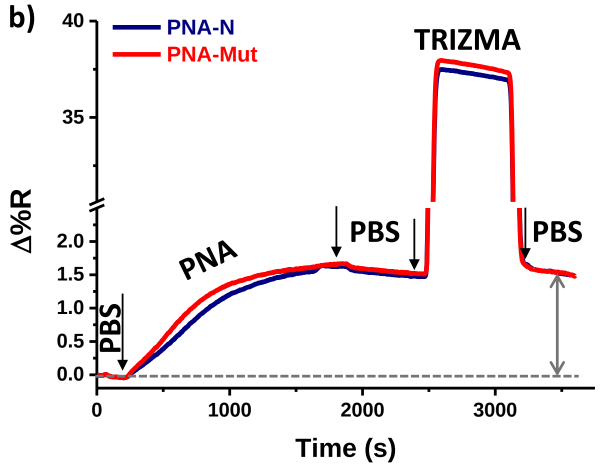

	Thickness (nm)	Surface coverage (molecules cm^{-2})
PNA-N	0.59 ± 0.12	(11.7 ± 2.3) x 10^{12}
PNA-M	0.59 ± 0.11	(11.8 ± 2.1) x 10^{12}

Figure S2. a) Δ%R over time detected for PNA-N or PNA-M probes multichannel parallel immobilization (0.05 µM, 20 min) on DTSP-SAM functionalized gold surface. The y-axis displays a breakpoint related to Trizma's refractive index variation (Δ%R = 31) to zoom in on the kinetic profile of PNAs recorded during their immobilization. Direct and different SPRI images have been acquired during the real-time monitoring of PNA probes anchoring. The same grey-scale has been obtained for both PNA-N and PNA-M, by indicating similar refractive index values at the immobilization reaction. b) Average kinetic curves of PNA-N and PNA-M recording during their immobilization for 44 replicates. The thickness and surface coverage of both PNA layers have also been shown.

Genomic DNA sequences extracted by healthy donors and beta-thalassemia patients

Extraction, purification, and characterization of genomic DNA (gDNA) samples were performed as described in the previous work.[4] Patients with classic β-thalassemia mutation in DNA sequences were identified by genotyping their β-globin gene by PCR amplification and DNA sequencing (Table S2).

Table S2. Quantification of genomic DNA sequences by PCR amplification from blood beta-thalassemic patients using for Dyna@B39-based plasmonic assay.

Thalassemia mutation	Genomic DNA (gDNA) sample ID	Stock solution concentration (ng µL^{-1})
$\beta^0$39 C>T homozygous, $\beta^0$39/ $\beta^0$39	Fe6	95.3
	Fe77	100.5
	pt#4	17.0

$\beta^0$39 C>T heterozygous, $\beta^0$39/WT	Fe18M	40.5
	Fe18M	41.2
$\beta^0$39 C>T heterozygous, $\beta^0$39/B^+IVSI-110	Fe30	56.2
WT/WT	FeWT1	127.5

Wild-type and $\beta^0$39 hetero/homo mutated gDNA were prepared at 1.5 pg µL^{-1} corresponding to 428 copies mL^{-1}, calculated by the following formula DNA copies mL^{-1} = $\frac{(DNA\, nanograms\ mL^{-1} * 6.0221 * 10^{23})}{(DNA\, base\, pairs * Molecular\, weight\ base\ pairs * 10^{9})}$, with DNA base pairs (bp) in genome human aploid equal to 3.2 10^9 and DNA bp molecular wieght equal to 660 g mol^{-1}), by considering a representative concentration of cfeDNA in SCM samples for embryo culture.

Calibration curve in medium blank

To assess the applicability of detecting low levels of cfeDNA for a non-invasive preimplantation genetic testing (niPGT) approach, we established a calibration curve across a dynamic range of target concentrations (0.5-20.0 pg µL^{-1}) of wild-type (WT/WT) gDNA spiked into the medium blank, utilizing the magnetic bead-based plasmonic assay (Figure S2 a,b). We determined the minimum detection concentration (MDC) and the reliable detection limit (RDL) using a four-parameter logistic curve fit. The four-parameter logistic equation for our experimental data is:

y = d + (a−d) / (1 + ([WT/WT gDNA] +2/c)^b)

In this equation, y represents the Δ%R ratio values (Δ%R PNA-N/Δ%R PNA-M) at various concentrations of spiked WT/WT gDNA [WT/WT gDNA] in medium blank. Figure S2 (b) displays the result of the four-parameter logistic fit (grey line, adj. R^2= 0.998) with the lower and upper 95% prediction limits (dashed grey lines). We determined MDC and RDL to be 0.41 pg µL^{-1} and 0.73 pg µL^{-1}, respectively. These values correspond to the interpolated intersections of the lower asymptote of the upper 95% prediction limit with the four-parameter logistic and the lower 95% prediction limit curves, respectively.

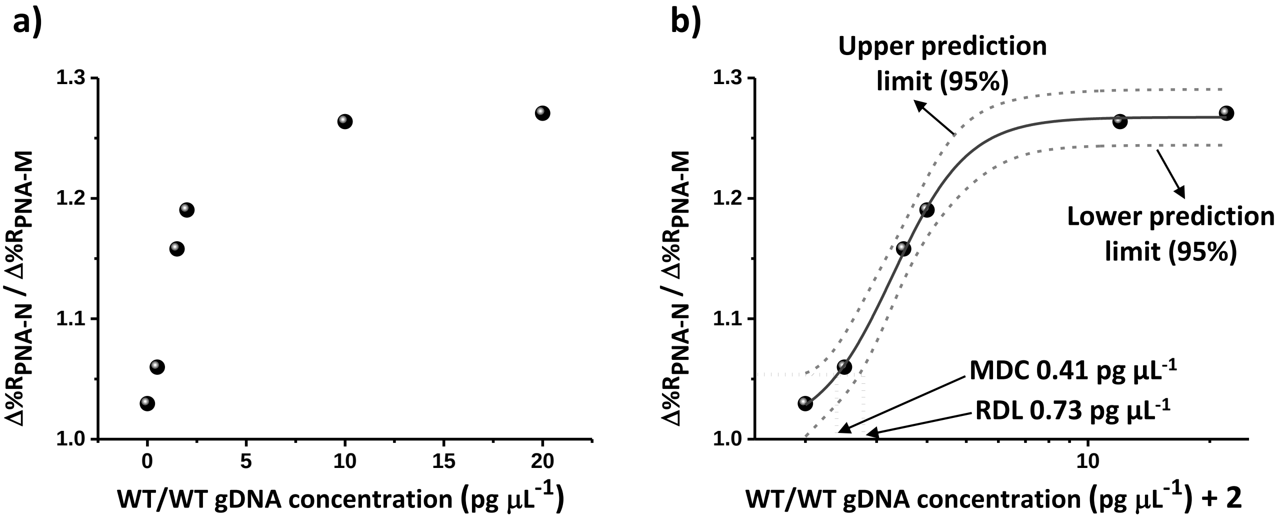

Figure S2. (a) SPRI calibration curve obtained from various concentrations of WT/WT gDNA spiked in medium blank added with 5% v,v of HSA. (b) Four-parameter logistic function fitting of the SPRI data (grey line). To incorporate the negative control (WT/WT gDNA concentration = 0) into the fitting procedure, we added 2 to the actual concentrations, in accordance with the method described in Ref. 6. This value was subsequently subtracted after the fitting process was completed. The regression (adj. R^2= 0.998) was obtained with the equation y = d + (a-d)/(1 + ([WT/WT gDNA] +2/c)^b) with parameters: a = -1.00554; b = 4.70264; c = 3.29279; d = 1.26736.

Embryo medium blank and spent culture medium samples

Table S3. Analysis of SCM samples collected from culture media of the embryos by trophectoderm biopsy and minisequencing method for the genotyping of $\beta^0$39 mutation. Preimplantation genetic testing (PGT) has been performed for monogenic disorder (PGT-M) and aneuploidy (PGT-A).

Sample ID	Sample type	Indications from PGT analysis	PGT results from embryos HBB Cod39 C>T
cfeGra2M	SCM from #embryo 2	PGT-M for β-thalassemia	WT/WT
cfeGra3M	SCM from #embryo 3	PGT-M for β-thalassemia	WT/WT
cfeGra4M	SCM from #embryo 4	PGT-M for β-thalassemia	WT/WT
cfeSam1M	SCM from *embryo 1	PGT-A	WT/WT
cfeSam2M	SCM from *embryo 2	PGT-A	WT/WT
cfeSam3M	SCM from *embryo 3	PGT-A	WT/WT
cfeSam4M	SCM from *embryo 4	PGT-A	WT/WT
cfeSam5M	SCM from *embryo 5	PGT-A	WT/WT
MBSam	Medium Blank	PGT-A	no cfeDNA
cfeGra1M	SCM from #embryo 1	PGT-M for β-thalassemia	heterozygous, $\beta^0$39/WT
cfeGra5M	SCM from #embryo 5	PGT-M for β-thalassemia	heterozygous, $\beta^0$39/WT

The symbols # and * denote various types of embryos, whereas the Arabic numbers indicate the quantity of embryos for the same category type.

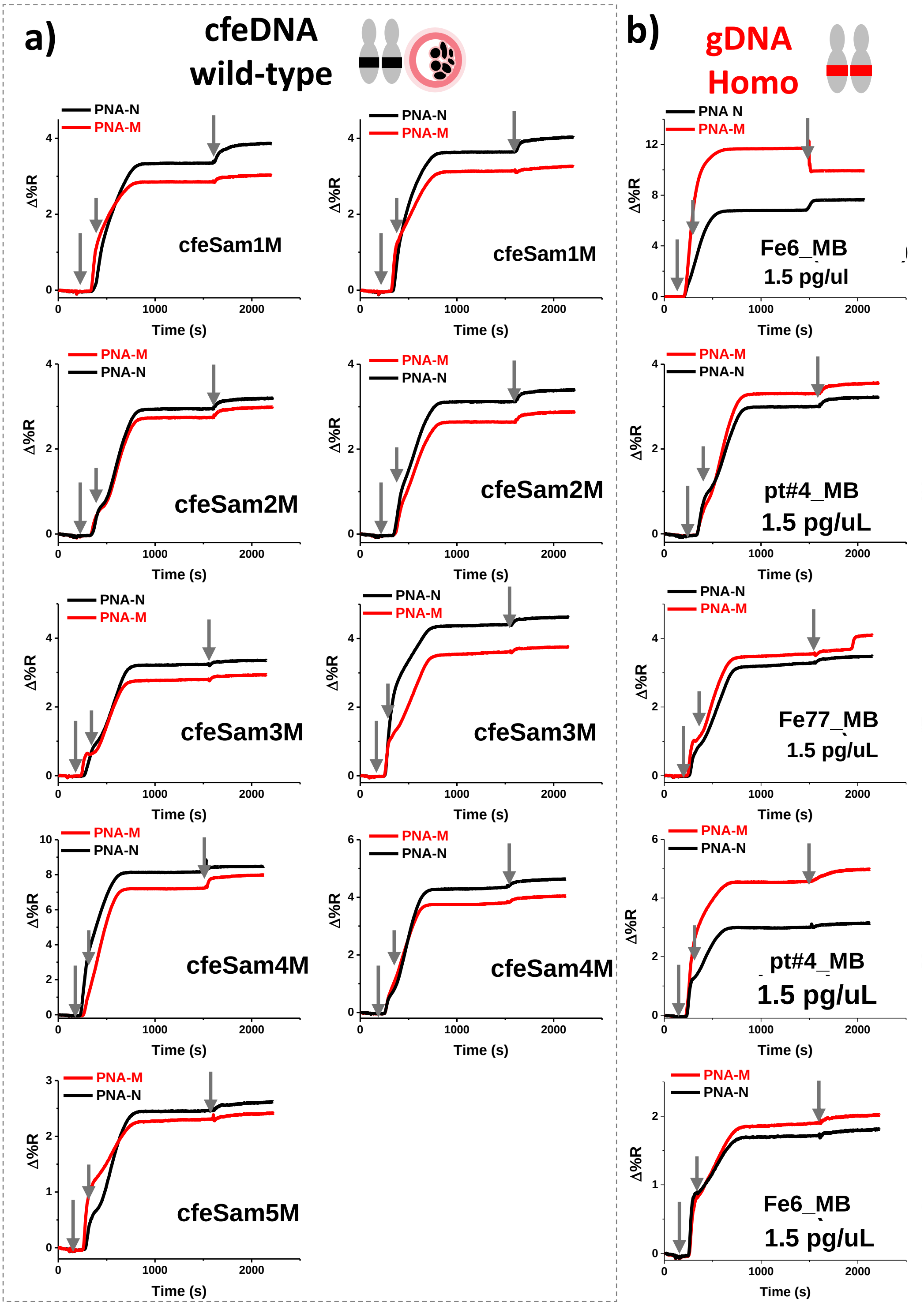

Figure S3. a) SPRI signals (Δ%R) over time for the hybridization of PNA-M and PNA-N probes with Dyna@B39-DNA bearing cfeDNA without $\beta^0$39 homo/hetero mutation (wild-type donors) captured in SCM of 5 embryos' cultures (cfeSam1M, cfeSam2M, cfeSam3M, cfeSam4M, and cfeSam5M) coming from the same parents. b) To compare the plasmonic signals, we performed the analysis of spiked $\beta^0$39 homo gDNA in MB plus 5% HSA in the same experiment. The analysis of sample cfeSam5M was not reproduced due to limitations in the sample size. As already described, the irregular shapes of curves shown in panels a and b were caused by the experimental procedure of the pumping system adopted to minimize the sample volume, as indicated by the grey arrows.

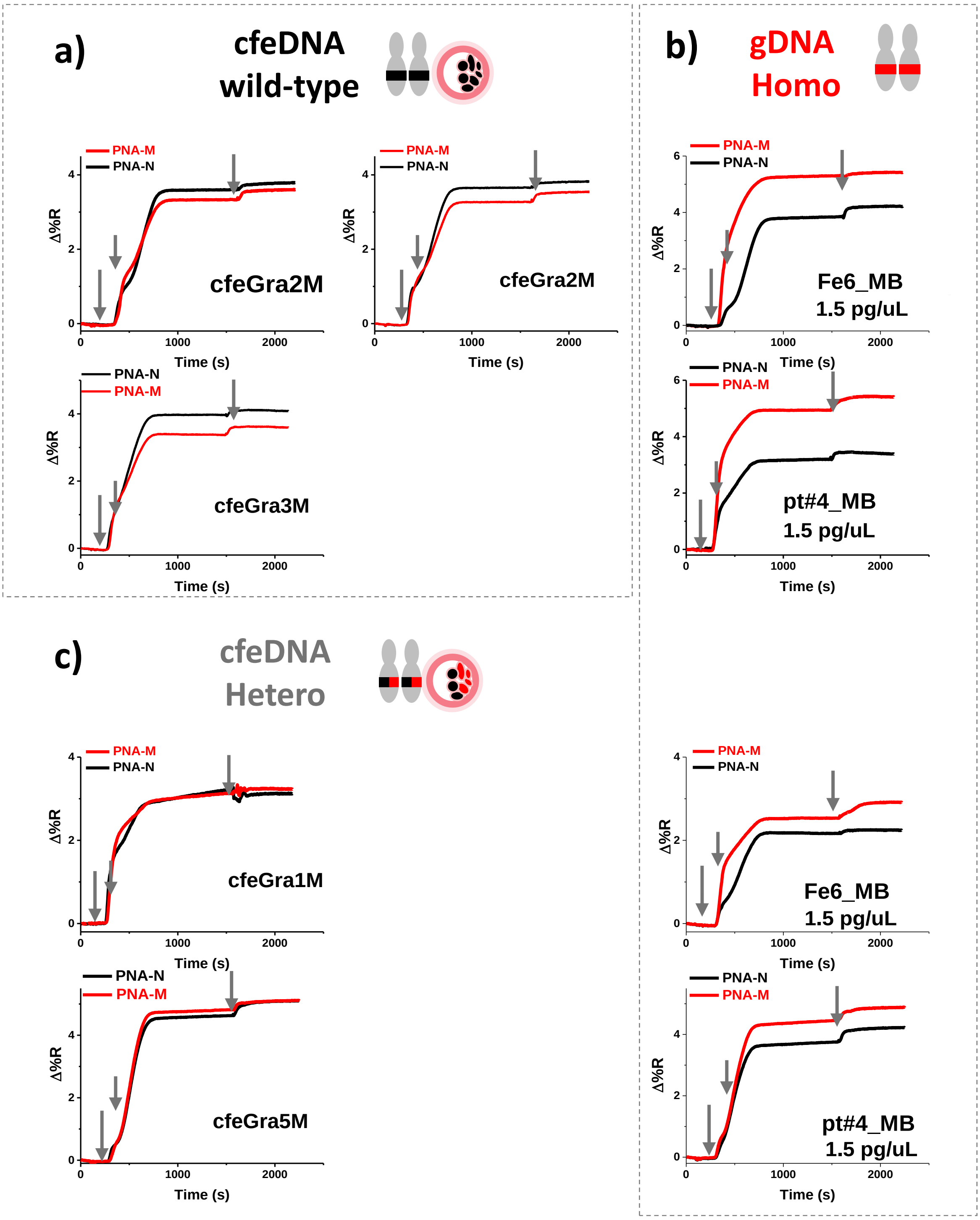

Figure S4. a) SPRI signals (Δ%R) over time for the hybridization of PNA-M and PNA-N probes with Dyna@B39-DNA bearing cfeDNA without $\beta^0$39 homo/hetero mutation (wild-type donors) captured in SCM of 2 embryos' cultures (cfeGra2M, cfeGra3M) coming from the same parents. b) To compare the plasmonic signals, we performed the analysis of spiked $\beta^0$39 homo gDNA in MB plus 5% HSA in the same experiment. c) SPRI signals (Δ%R) over time for the hybridization of PNA-M and PNA-N probes with Dyna@B39-DNA bearing cfeDNA with $\beta^0$39 hetero mutation captured in SCM of 2 embryos' cultures (cfeGra1M, cfeGra5M) coming from the same parents. The analysis of sample cfeGra1M, cfeGra3M and cfeGra5M was not reproduced due to limitations in the sample size. As already described, the irregular shapes of curves shown in panels a, b, c were caused by the experimental procedure of the pumping system adopted to minimize the sample volume, as indicated by the grey arrows.

Table S4. Population mean confidence intervals of $\Delta\%R_{PNA-M}/\Delta\%R_{PNA-N}$ ratios for analyzed SCM, with ID of samples used for the analysis and the number of replicates. The mean $\Delta\%R_{PNA-M}/\Delta\%R_{PNA-N}$ ratio value is reported in Fig. 4.

Genetic status	Sample ID	Mean $\Delta\%R_{PNA-M}/\Delta\%R_{PNA-N}$	Replicates
cfeDNA wild-type	cfeSam1M	0.803 ± 0.009	2
	cfeSam2M	0.89 ± 0.06	2
	cfeSam3M	0.84 ± 0.04	2
	cfeSam4M	0.90 ± 0.04	2
	cfeSam5M	0.92	1
	cfeGra2M	0.92 ± 0.02	1
	cfeGra3M	0.88	1
cfeDNA $\beta^0$39 hetero	cfeGra1M	0.99	1
	cfeGra5M	1.00	1
gDNA $\beta^0$39 homo spiked in medium blank	Fe6_MB	1.3 ± 0.2	4
	pt#4_MB	1.3 ± 0.2	4
	Fe77_MB	1.06	1

References

(1) Bellassai, N.; D'Agata, R.; Spoto, G. Plasmonic Aptasensor with Antifouling Dual-Functional Surface Layer for Lysozyme Detection in Food. *Anal Chim Acta* **2023**, *1283*, 341979. https://doi.org/10.1016/j.aca.2023.341979.

(2) Giesen, U.; Kleider, W.; Berding, C.; Geiger, A.; Orum, H.; Nielsen, P. E. A Formula for Thermal Stability (Tm) Prediction of PNA/DNA Duplexes. *Nucleic Acids Res* **1998**, *26* (21), 5004–5006. https://doi.org/10.1093/nar/26.21.5004.

(3) Germini, A.; Rossi, S.; Zanetti, A.; Corradini, R.; Fogher, C.; Marchelli, R. Development of a Peptide Nucleic Acid Array Platform for the Detection of Genetically Modified Organisms in Food. *J Agric Food Chem* **2005**, *53* (10), 3958–3962. https://doi.org/10.1021/jf050016e.

(4) D'Agata, R.; Breveglieri, G.; Zanoli, L. M.; Borgatti, M.; Spoto, G.; Gambari, R. Direct Detection of Point Mutations in Nonamplified Human Genomic DNA. *Anal Chem* **2011**, *83* (22), 8711–8717. https://doi.org/10.1021/ac2021932.

(5) Shumaker-Parry, J. S.; Campbell, C. T. Quantitative Methods for Spatially Resolved Adsorption/Desorption Measurements in Real Time by Surface Plasmon Resonance Microscopy. *Anal Chem* **2004**, *76* (4), 907–917. https://doi.org/10.1021/ac034962a.

(6) Holstein, C. A.; Griffin, M.; Hong, J.; Sampson, P. D. Statistical Method for Determining and Comparing Limits of Detection of Bioassays. *Anal Chem* **2015**, *87* (19), 9795–9801. https://doi.org/10.1021/acs.analchem.5b02082.
